# Investigation of Polyphenolic Compounds in Different Varieties of Black Chokeberry *Aronia melanocarpa*

**DOI:** 10.3390/molecules28104101

**Published:** 2023-05-15

**Authors:** Makar A. Gerasimov, Irina B. Perova, Konstantin I. Eller, Michail Y. Akimov, Anna M. Sukhanova, Galina M. Rodionova, Galina V. Ramenskaya

**Affiliations:** 1 Federal Research Centre of Nutrition, Biotechnology and Food Safety, 2/14, Ustinsky Proezd, 109240 Moscow, Russia; 2 Federal State Autonomous Educational Institution of Higher Education I.M. Sechenov First Moscow State Medical University of the Ministry of Health of the Russian Federation (Sechenov University), 8-2 Trubetskaya Str., 119991 Moscow, Russia; 3 I.V. Michurin Federal Scientific Center, Federal State Scientific Institution, 30, Muchurin St., Tambov Region, 393774 Michurinsk, Russia

**Keywords:** black chokeberry, anthocyanins, proanthocyanidins, flavonoids, hydroxycinnamic acids

## Abstract

The purpose of this work was to study the qualitative and quantitative composition of the main groups of biologically active substances in the fresh fruits of five different varieties of black chokeberry (*Aronia melanocarpa* (Michx.) Elliot), carried out within the framework of the search for available and cost-effective raw materials for food product fortification. Samples of aronia chokeberry were grown at the Federal Scientific Center named after I.V. Michurin in the Tambov region of Russia. Using a modern chemical–analytical methodology, the contents and profiles of anthocyanin pigments, proanthocyanidins, flavonoids, hydroxycinnamic acids, organic acids (malic, quinic, succinic, and citric), monosaccharides, disaccharides, and sorbitol were determined in detail. Based on the results of the study, the most promising varieties were determined in terms of the content of the main biologically active substances.

## 1. Introduction

Aronia chokeberry (*Aronia melanocarpa* (Michx.) Elliot) is a perennial shrub of the Rosaceae family that is native to the eastern regions of North America. It was brought to Europe at the beginning of the 20th century. Aronia is cultivated all over the world, but it has gained the greatest popularity in the northern (Denmark, Estonia, Latvia, Lithuania, and Sweden), eastern (Bulgaria, Poland, and Serbia), and central (Czech Republic and Germany) countries of Europe. Poland is the world’s largest producer of chokeberries, accounting for almost 90% of the world’s production [1]. According to the data from the State Census of Gardens in 1970, there were more than 5500 hectares of chokeberry plantations in the USSR or more than 6 million bushes. Today, in Russia, the black chokeberry occupies about 1000 hectares. The industrial plantings are located mostly in the Altai and Kaluga regions. In the Tambov region, farmers of the I.V. Michurin Federal Scientific Center have also begun to cultivate black chokeberry on an industrial scale in the black earth zone. The current area of these gardens already exceeds 50 hectares, and it is planned to increase it to 75 hectares. Good prospects for the development of culture and the increase in industrial areas are associated with a wide range of positive biological properties and technological characteristics of black chokeberries, such as precocity, a high and fairly stable yield, good winter hardiness, and resistance to pests and diseases.

This species is highly adaptable and grows in a wide variety of conditions. Due to its strong cold resistance, in addition to its ability to grow in mild climatic conditions, the crop can be grown at temperatures below −35 °C [2]. It is easily propagated from seeds, gives a high yield, and is therefore economically viable.

Aronia is a rich source of phenolic compounds such as anthocyanins, chlorogenic acids, flavonols, and proanthocyanidins, which have strong antioxidant potential. According to the *Pharmocopoeia of the Russian Federation*, the standardization of fresh and dry *Aronia melanocarpa* fruits is carried out based on the amount of anthocyanins in terms of cyanidin-3-*O*-glucoside [3].

Juices, tablets, and tonics are made from chokeberry fruits for medicinal purposes. The juices and tonics are used to treat hypertension and atherosclerosis, while the tablets are taken as a vitamin remedy. It has been experimentally proven that chokeberry has anti-inflammatory, antitumor, antimicrobial, antiviral, antiatherosclerotic, antiplatelet, hypotensive, antidiabetic, and anti-inflammatory properties, which are directly related to the antioxidant capacity of the polyphenolic compounds [4,5,6,7,8]. To date, black chokeberry has found a wide range of applications in the food industry, both: as a source of natural food coloring (in juices, jams, wine, and liqueurs) and as a rich source of phytonutrients for dietary supplements and fortified foods with a wide range of beneficial effects [9].

## 2. Results

### 2.1. Anthocyanins

**Figure 1:**
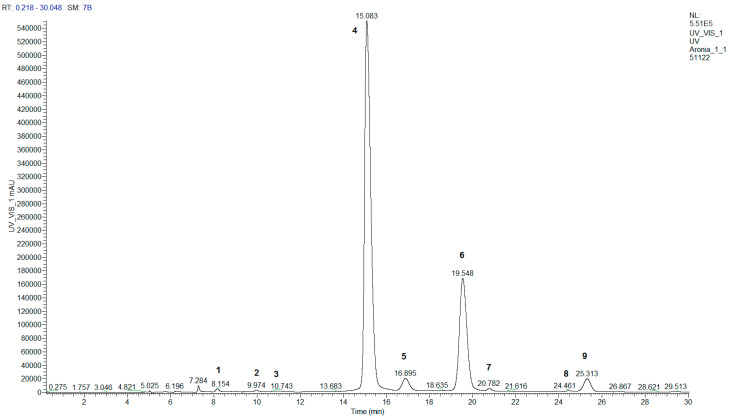
HPLC–DAD chromatogram of “Nadzeya” chokeberry fruit extract at 520 nm. The numbers of anthocyanin peaks in the chromatogram correspond to those in Table 1.

The anthocyanins identified in chokeberry fruits using HPLC–DAD–MS are shown in Figure 1 and described in Table 1. Identification of peaks was carried out according to retention time, elution order, UV-vis spectra, and mass spectra by comparison with literature data. Along with the four main chokeberry anthocyanins (cyanidin-3-galactoside, cyanidin-3-glucoside, cyanidin-3-arabinoside, and cyanidin-3-xyloside), some minor ingredients, such as three anthocyanin flavanol condensation products and two pyranoanthocyanins, were detected. Similarly, cyanidin-3-hexoside-epicatechin, cyanidin-3-pentoside-epicatechin, and cyanidin-3-hexoside-epicatechin-epicatechin were previously found in “Galichanka” chokeberry fruits cultivated in Poland [10]. The pyranoanthocyanins 5-carboxypyranocyanidin-3-hexoside and 5-carboxypyranocyanidin-3-pentoside were identified for the first time. Pyruvates of anthocyanins are usually formed in berries during storage and/or processing. Previously, we found 5-carboxypyranomalvidin-3-glucoside (vitisin A), pyranomalvidin-3-glucoside (vitisin B), 5-carboxypyranocyanidin-3-glucosylrutinoside, 5-carboxypyranocyanidin-3-rutinoside, and some other pyranoanthocyanins in grape juices and sour cherry nectars but not in the corresponding fresh and frozen berries. Nevertheless, cyanidin-3-glycoside-epicatechin derivatives and 5-carboxypyranocyanidin-3-glycosides combined ranged between 0.7 and 1.0% of the total anthocyanins (TAC) in chokeberries (Table 2) and consequently contributed insignificantly to biological activity. Two major anthocyanins were cyanidin-3-galactoside (65.6–69.3% of TAC) and cyanidin-3-arabinoside (24.2–26.9% of TAC). The average ratio of the two major anthocyanin concentrations was 2.44–2.86.

The lowest TAC content was found in fruits from the cultivar “Cherno-plodnaya” (388.1 mg C3GlE/100 g FW); in the fruits of the other four cultivars, TAC quantities ranged between 590.0 in “Venisa” and 620.5 mg C3GlE/100 g FW in “Aron”. According to the published data, TAC concentrations in chokeberries varied in a wide range from 192.2 to 2134.9 mg per 100 g of FW [10,11,12,13,14,15,16,17,18,19]. Such a wide range of anthocyanin concentrations may be due to various factors, including cultivar, genotype, growing area, maturity, harvest date, extraction procedure, and even method of analysis (direct UV–spectrophotometry, pH differential method, HPLC–DAD, or HPLC–MS). In general, chokeberry should be considered one of the richest sources of anthocyanins among berries.

### 2.2. Proanthocyanidins

Proanthocyanidins (PACs) are the largest group of polyphenols in chokeberries and are mostly responsible for the fruit’s astringent taste. PACs, along with monomeric anthocyanins, determine the biological activity of chokeberries to a great extent. Total PAC (TPAC) contents in mg procyanidin B2 equivalents (PCB2E) per 100 g FW are shown in Table 3. It was found that epicatechin was the major monomeric unit of chokeberry PACs. Free epicatechin was detected in all varieties at low concentrations ranging from 1.7 to 8.3 mg/100 g FW (Table 3). Trace amounts of catechin were detected only in the cultivars “Cherno-plodnaya” and “Mulatka”. Previously, higher concentrations of epicatechin (15.8–109.4 mg/100 g FW) and catechin (21.5 mg/100 g FW) were found in black chokeberries from Poland, the Czech Republic, and Bulgaria [10,13,15,18]. “Cherno-plodnaya” fruit contained the lowest amount of TPAC (1395.8 mg PCB2E/100 g FW) among the studied samples, and the fruits of the four other cultivars showed TPAC contents in a narrow range between 2198.9 in “Aron” and 2524.1 mg PCB2E/100 g FW in “Nadzeya”. The same tendency as for TAC content was observed for TPAC content. The TPAC quantities obtained in this work are similar to those reported by Wangensteen (2.46–3.74 g PCB2E/100 g FW) [11], and approximately two times higher than the polymeric PAC quantities (1426.6–1751.2 mg/100 g FW) estimated using phloroglucinolysis–HPLC–FLD and thiolysis–HPLC–DAD–FLD [10,15]. In terms of PAC profile, only two B-type dimmers, B2 and B5, with [M + H]^+^/[M − H]^−^ 579/577, and one trimer, C1, with [M + H]^+^/[M − H]^−^ 867/865, were found, and only in negligible quantities. Procyanidin B2 was identified by comparison with an authentic analytical standard, whereas procyanidins B5 and C1 were identified using mass spectrometry in accordance with literature data [14]. Oligomeric PACs from tetramers to hexamers were not detected in any of the five varieties. This indicates that the chokeberry PAC fraction consists predominantly of higher oligomers with a polymerization degree greater than C6 and polymers, as confirmed in [10,14] and by several other researchers [9,20].

### 2.3. Flavonols

**Figure 2:**
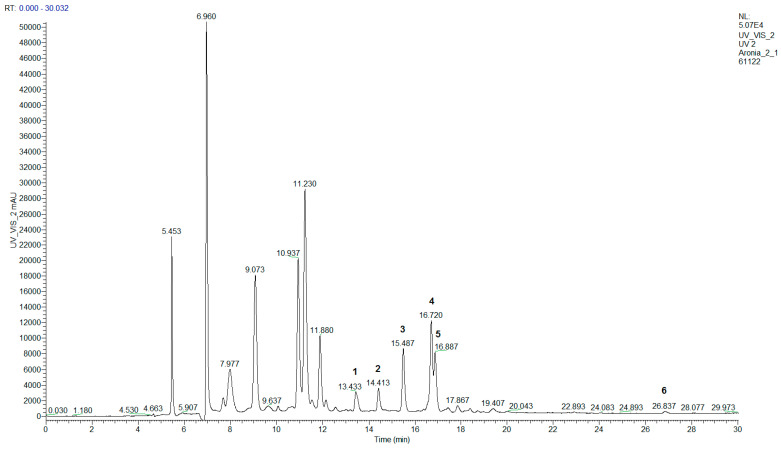
HPLC–DAD chromatogram of “Aron” chokeberry fruit extract at 350 nm. The numbers of flavonol peaks in the chromatogram correspond to those in Table 4.

In terms of flavonols, only quercetin derivatives were found: five glycosides and free quercetin (Figure 2, Table 4). Rutin, hyperoside, isoquercitrin, and quercetin were identified against the corresponding authentic analytical standards. Tentative identification of dihexoside and 3-vicianoside of quercetin was achieved by comparison of elution order, UV-vis, and mass spectra with literature data [21]. According to the literature data, chokeberry flavonols include mostly quercetin glycosides, although the presence of isorhamnetin and kaempferol glycosides has also been reported [9,10,11,12,13].

The three major flavonol glycosides in all chokeberries were hyperoside, rutin, and isoquercitrin (Table 5). Rutin was the predominant flavonol glycoside only in fruit from the cultivar “Cherno-plodnaya”, whereas, in the fruits from the other four cultivars, hyperoside was predominant. The contents of quercetin-dihexoside and quercetin-3-vicianoside were in the range of approximately 1.5–3.4 times less than the hyperoside, rutin, and isoquercitrin contents, except in “Cherno-plodnaya” fruits, in which approximately equal quantities of quercetin-3-vicianoside and isoquercitrin were found. Quercetin was a minor component in all samples. Total flavonol contents ranged from 21.3 mg/100 g FW in “Cherno-plodnaya” fruits to 58.1 mg/100 g in “Aron” fruits. In various published studies, the three main flavonol glycosides in different cultivars of black chokeberries have been reported to be the same as in our samples [5,11]. Similar results for total flavonol contents (19.2–55 mg/100 g FW) were also obtained by many other researchers [11,12,15]. At the same time, several authors observed higher amounts of total flavonols (64.8–124.9 mg/100 g FW) in black chokeberry cultivars from Bulgaria and Poland [10,11,12,13,22].

### 2.4. Hydroxycinnamic Acids (HCAs)

HCAs were represented by caffeoylquinic acid isomers: neochlorogenic, chlorogenic, and cryptochlorogenic acids (Table 6). The identification of neochlorogenic and chlorogenic acids was carried out with the use of authentic analytical standards, whereas cryptochlorogenic acid was identified using elution order, UV-vis spectra, and mass spectrometry. Concurrently, dicaffeoylquinic acid, *p*-coumaroylquinic, caffeoylshikimic acid isomers, sinapic acid, and HCA’ glucosides, identified as minor components in some chokeberries by several researchers [9,10,11], were not detected in this work.

Chlorogenic acid was the major HCA in all varieties and accounted for 51.8–60.8% of the total HCA content. The neochlorogenic acid amount was 1.3–1.8 times less than that of chlorogenic acid. Cryptochlorogenic acid was a minor HCA in all samples.

Total HCA contents ranged from 41.1 mg/100 g FW in “Venisa” fruits to 136.9 mg/100 g in “Nadzeya” fruits. These results agreed with the results obtained by many other authors [11,12,23]. In contrast, significantly higher total HCA contents in black chokeberries of different cultivars from the Czech Republic, Poland, and Bulgaria ranged from 189.9 to 407.0 mg/100 g FW, as was previously reported by Rop, Tarko, Denev, Oszmiański, and Skupien [10,13,15,18].

### 2.5. Total Polyphenols and Antiradical Activity

The results of the experiment showed great variability in total polyphenol (TP) contents among the analyzed samples (Table 7). The lowest TP amount was found in “Cherno-plodnaya” fruits (747.2 mg GAE/100 g FW), and much higher TP amounts were found in the four other cultivars’ fruits, ranging from 1369.9 in “Nadzeya” to 1667.8 mg GAE/100 g FW in “Mulatka”. TP concentrations corresponded primarily to those of TAC and, to a lesser degree, with those of flavonols and HCAs. The contribution of TAC to TP content was smallest in “Mulatka” fruits (36.9%) and greatest in “Cherno-plodnaya” fruits (51.9%). Previously published data about TP quantities in different chokeberries are also very variable, ranging from 603 to 2377.1 mg GAE/100 g FW [11]. Certain factors, including cultivar, growing area, climatic conditions, extraction procedure, and Folin–Ciocalteu method modification, can explain this wide difference in TP results.

In the case of DPPH–radical scavenging activity, the same tendency was observed for TAC, TPAC, total flavonols, and TP content. “Cherno-plodnaya” was the cultivar with the weakest antiradical activity (523.8 mg TE/100 g FW), whereas the four other cultivars showed stronger antiradical activities ranging from 1001.5 mg/100 g in “Nadzeya” to 1188.1 mg/100 g FW in “Aron”. A correlation was found between the contents of the two main groups of polyphenols (anthocyanins and proanthocyanidins), TP content, and antiradical activity in the analyzed samples.

### 2.6. Organic Acids

The contents of sugars and organic acids are directly related to a fruit’s taste properties and nutritional value. Detailed information on the contents of organic acids can be found in Table 8.

As can be seen from Table 8, malic acid was the main organic acid in the studied varieties of chokeberry. Its content ranged from 55 to 59% of the sum of all organic acids. Denev et al. [13] provide evidence that quinic acid predominates in some varieties of black chokeberry. According to our data, quinic acid has a fairly high level of accumulation of up to 38%, but the highest content was accounted for by malic acid, which, in turn, is consistent with the results of other studies [11,18]. We also noted a relatively high concentration of succinic acid, which is unusual for fresh chokeberry fruits.

### 2.7. Sugars and Sorbitol

Our results for the contents of the main macrocomponents are as follows: L-malic acid (600–1500 mg/100 g), citric acid (20–70 mg/100 g), quinic acid (200–700 mg/100 g), shikimic acid (max 15 mg/100 g), and sorbitol (4–6 g/100 g). These results closely correspond with the appropriate AIJN specification for aronia puree/juice. For fructose and glucose (Table 9), our data were slightly higher than the current AIJN specification (glucose 3–4 g/100 g, fructose 2.5–3.8 g/100 g). It was interesting to note the presence of succinic acid in all varieties, the indicator of which is not regulated by AIJN.

## 3. Discussion

In terms of biological value, the main groups of polyphenols in chokeberries are anthocyanins and proanthocyanidins. Conversely, flavonols, HCAs, and catechins should be considered the minor groups of polyphenols with less substantial contributions to biological activity. Among the analyzed samples, “Cherno-plodnaya” fruit was distinguished by the lowest amounts of TACs, TPACs, flavonols, and TPs and the weakest antiradical activity. Fruits of the other four cultivars contained approximately equal concentrations of TACs and TPACs; concurrently, “Nadzeya” and “Aron” fruits had higher levels of flavonols and HCAs. Thus, the chokeberries “Nadzeya”, “Aron”, “Venisa”, and “Mulatka” are suitable for use both for pharmaceutical purposes and in the food industry for the production of fortified foods and dietary supplements.

Thanks to the results obtained, it is possible to select more promising regional cultivars for potential use in the food and pharmaceutical industries, as well as for further genetic crossing.

## 4. Materials and Methods

### 4.1. Plant Material

Five chokeberry cultivars of different origins were investigated: *Aronia melanocarpa* “Nadzeya” (Belarus), “Cherno-plodnaya” (Russia), “Venisa” (Belarus), “Mulatka” (Russia), and *Aronia prunifolia* “Aron” (Denmark). The plant material was cultivated and gathered at the I.V. Michurin Federal Scientific Center (Tambov region, Michurinsk) in September 2022. Fresh chokeberries were frozen and kept at −18 °C before extraction.

### 4.2. Reagents and Solvents

For the extraction and analysis of polyphenol compounds, organic acids, and sugars, the following reagents and solvents were used: formic acid (98.0–100%, Sigma-Aldrich, St. Louis, MO, USA), hydrochloric acid (chemically pure, Chimmed, Moscow, Russia), potassium chloride (chemically pure, Vekton, Saint Petersburg, Russia), sodium acetate trihydrate (pure, Chemreactive, Nizhniy Novgorod, Russia), potassium metabisulfite (chemically pure, Prime Chemicals Group, Mytishshi, Russia), ammonium iron(III) sulfate dodecahydrate (reagent grade, Prime Chemicals Group, Russia), sodium carbonate anhydrous (chemically pure, Vekton, Russia), Folin–Ciocalteu reagent (Sigma-Aldrich, USA), casein (92%, J&K Scientific, San Jose, CA, USA), DPPH (Sigma-Aldrich, USA), ultrapure water (Milli-Q^®^, Merck Millipore, St. Louis, MO, USA), methanol (HPLC gradient grade, J.T. Baker, Landsmeer, Holland), ethanol (95%, Constanta Pharm M, Moscow, Russia), acetonitrile (for UV, IR, HPLC, ACS, PanReac Applichem, Barcelona, Spain), and 1-butanol (chemically pure, Vekton, Russia). Identification and quantification of biologically active substances were carried out using pure substances: procyanidin B2 (≥90%, INDOFINE Chemical Company, Hillsborough, NJ, USA), rutin trihydrate (≥95%, Roth, Germany), hyperoside (≥95%, HWI ANALYTIK GMBH, Rulzheim, Germany), isoquercitrin (≥94%, HWI ANALYTIK GMBH, Germany), quercetin hydrate (≥95%, Sigma-Aldrich, USA), (+)-catechin hydrate (95%, ABCR, Karlsruhe, Germany), (−)-epicatechin (95%, ABCR, Germany), neochlorogenic acid (≥98%, Sigma-Aldrich, USA), chlorogenic acid (≥95%, Sigma-Aldrich, USA), gallic acid (98%, J&K Scientific, USA), trolox (≥97%, Aldrich, Søborg, Denmark), *D*-(+)-glucose (≥99.5%, Sigma-Aldrich, USA), *D*-sorbitol (≥98%, Sigma-Aldrich, USA), *D*-(−)-fructose (≥99%, Sigma-Aldrich, USA), sucrose (99.5%, Sigma-Aldrich, USA), *D*-(−)-quinic acid (98%, J&K Scientific, USA), citric acid (99%, Sigma-Aldrich, USA), *L*-(−)-malic acid (≥99%, Sigma-Aldrich, USA), succinic acid (≥99%, Sigma-Aldrich, USA), shikimic acid (≥99%, Sigma-Aldrich, USA), *L*-(+)-ascorbic acid (≥99%, Alfa Aesar, Haverhill, MA, USA).

### 4.3. Extraction Procedure

Frozen chokeberries were crushed in a mortar until pureed. Pureed samples (5 g) were extracted using 70% aqueous ethanol acidified with 0.1 N HCl. The extraction was carried out three times (40 mL, 30 mL, and 20 mL) by sonication (Bandelin RK 31, BANDELIN electronic GmbH & Co. KG, Berlin, Germany) at room temperature (RT) for 15 min. Each extract was centrifuged at 14,000× *g* for 10 min. Three supernatants were combined in a volumetric flask of 100 mL, diluted with a solvent to volume, and stirred. The 70% ethanol extract obtained was used for total anthocyanin and anthocyanin profile analysis. Total proanthocyanidins, total polyphenols, flavonol and HCA profiles, and antiradical activity were determined in aqueous methanol extracts. Pureed samples (5 g) were placed into round-bottomed 100-mililiter flasks, and 50 mL of 60% aqueous methanol was added. Extraction was performed by heating in a boiling-water bath (GFL 1031, GFL Gesellschaft für Labortechnik mbH, Burgwedel, Germany) for 1 h. Next, the extract was cooled to RT and centrifugated at 14,000× *g* for 10 min. The supernatant was transferred into a volumetric flask of 50 mL, diluted to volume with 60% aqueous methanol, and stirred. Extract aliquots were additionally filtered through a hydrophilic PTFE 0.22 µm membrane (Millex^®^, Merck, Darmstadt, Germany) into autosampler screw-cap vials before HPLC–DAD–MS analysis.

### 4.4. UV Measurements

A Shimadzu UV 1800 spectrophotometer (Shimadzu Corporation, Kyoto, Japan) was used to measure the absorbance of the analyzed solutions.

#### 4.4.1. Total Anthocyanins

Total monomeric anthocyanins expressed as cyanidin-3-glucoside equivalents were determined via the pH differential method (AOAC 2005.02).

#### 4.4.2. Total Proanthocyanidins

Total proanthocyanidins expressed as procyanidin B_2_ equivalents were determined using the modified Bate–Smith method [24]. Acid butanol hydrolysis: screw-cap vials containing 3 mL of a mixture of butanol—12 N HCl 95:5 (*v*/*v*), 0.1 mL of 2% FeNH_4_(SO_4_)_2_ solution in 2 N HCl, and 0.5 mL of procyanidin B_2_ calibration solution, sample solution, or solvent (for blank solution) were heated to 95 °C for 45 min. Vials with the mixture were then cooled to RT, and the absorbance was read at 550 nm. The procyanidin B_2_ calibration curve was constructed at a concentration range of 0.01–0.3 mg/mL (R^2^ = 0.9997).

#### 4.4.3. Total Polyphenols

Total polyphenol contents expressed as gallic acid equivalents (GAE) were estimated using the Folin–Ciocalteu method [25]. Sample solutions were diluted 10 times with extraction solvent (60% aqueous methanol). Screw–cap vials containing 1 mL of gallic acid calibration solution or diluted sample solution, 5 mL of 10% Folin–Ciocalteu reagent solution, and 4 mL of 7.5% sodium carbonate solution were stored in the dark at RT for 1 h. The absorbance of the mixture was read at 765 nm, and distilled water was used as the blank solution. The gallic acid calibration curve was prepared at a concentration range of 0.01–0.09 mg/mL (R^2^ = 0.9997).

#### 4.4.4. Free Radical Scavenging Activity towards DPPH Radicals

Free radical scavenging activity expressed as Trolox equivalents (TE) was determined via DPPH radical decolorization as described by Brand-Williams et al. [26]. To 3 mL of DPPH radical solution, 0.04 mg/mL (A_515_ 1.0), 15 µL of Trolox calibration solution, sample solution, or extraction solvent (for blank solution) were added. The mixture was stirred and stored in the dark at RT for 30 min. The absorbance of the mixture was then measured at 515 nm. The Trolox calibration curve was created at a concentration range of 0.005–0.15 µg/15 µL (R^2^ = 0.9998).

### 4.5. Determination of Anthocyanin, Flavonol, Catechin, and HCA Profiles Using HPLC–DAD–MS

Thermo Scientific Ultimate 3000 rapid separation dual system equipped with a diode array detector (DAD) and TSQ Endura Triple-Stage Quadrupole mass spectrometer (MS) was used for identification and quantification of polyphenol compounds (anthocyanins, flavonols, catechins, and hydroxycinnamic acids) in chokeberries. HPLC–DAD–MS conditions are described in Table 10.

Calibration curves of rutin, hyperoside, isoquercitrin, quercetin, neochlorogenic and chlorogenic acids, catechin, and epicatechin were constructed at concentration ranges of 0.005–0.25 mg/mL (R^2^ > 0.999). Data handling was carried out using Thermo Xcalibur (Thermo Fisher Scientific Inc. Waltham, MA, USA) 4.2.47 software.

### 4.6. Determination of Monosaccharides, Disaccharides, and Sorbitol via Capillary Electrophoresis with DAD

An Agilent 7100 capillary electrophoresis system equipped with DAD was used for monosaccharide, disaccharide, and sorbitol analysis. Separation took place on a bare fused silica capillary with an effective length of 72 cm and an internal diameter of 50 µm at 15 °C using a basic anion buffer [27]. Indirect UV detection was carried out at 350 nm with a reference of 275 nm. Glucose, fructose, saccharose, and sorbitol calibration curves were constructed at concentration ranges of 0.05–10.0 mg/mL (R^2^ > 0.999).

### 4.7. Determination of Organic Acids Using HPLC–VWD

An Agilent 1100 HPLC system equipped with an isocratic pump and variable wavelength detector (VWD) was used for organic acid analysis. Separation of organic acids was carried out on a Phenomenex Luna C18 150 × 4.6 mm with a particle size of 5 µm LC column by elution with a 0.1 M KH_2_PO_4_ buffer solution (pH 2.4) at 25 °C. The flow rate was 1.0 mL/min, the injection volume was 20 µL, and the VWD was set at 210 nm. Quinic, malic, citric, succinic, and shikimic acid calibration curves were created at concentration ranges of 0.05–1.0 mg/mL (R^2^ > 0.999).

All measurements were performed in triplicate, and results are presented in mg per 100 g FW.

## Figures and Tables

**Table 1:** Identification of chokeberry anthocyanins using HPLC–DAD–MS.

No.	Anthocyanin	Rt, min	λ_max_, nm	HESI–MS^+^
1	Cyanidin-3-hexoside-epicatechin	8.1	524, 280	737.30 [M]^+^, 287.21 [M − hexose * − epicatechin]^+^
2	Cyanidin-3-pentoside-epicatechin	9.9	520, 280	707.31 [M]^+^, 287.20 [M − pentose − epicatechin]^+^
3	Cyanidin-3-hexoside-epicatechin- epicatechin	10.7	520, 280	1025.49 [M]^+^, 287.21 [M − hexose − epicatechin − epicatechin]^+^
4	Cyanidin-3-galactoside	15.0	516, 280	449.20 [M]^+^, 287.14 [M − galactose]^+^
5	Cyanidin-3-glucoside	16.9	516, 280	449.20 [M]^+^, 287.14 [M − glucose]^+^
6	Cyanidin-3-arabinoside	19.5	516, 280	419.18 [M]^+^, 287.14 [M − arabinose]^+^
7	5-carboxypyranocyanidin-3-hexoside	20.7	504, 280	517.25 [M]^+^
8	5-carboxypyranocyanidin-3-pentoside	24.4	508, 280	487.21 [M]^+^
9	Cyanidin-3-xyloside	25.2	516, 280	419.18 [M]^+^, 287.16 [M − xylose]^+^

* Here and in subsequent tables: dehydrated (–H_2_O) sugar moiety, because water is lost upon glycosidic bond formation.

**Table 2:** Anthocyanin profiles and total anthocyanin contents in chokeberries.

No.	Anthocyanin	“Nadzeya”	“Aron”	“Cherno- Plodnaya”	“Venisa”	“Mulatka”
	Anthocyanin profile, % from TAC					
1	Cyanidin-3-hexoside-epicatechin	0.3 ± 0.02	0.3 ± 0.01	0.2 ± 0.01	0.2 ± 0.01	0.2 ± 0.02
2	Cyanidin-3-pentoside-epicatechin	0.2 ± 0.01	0.1 ± 0.02	0.2 ± 0.02	0.1 ± 0.01	0.1 ± 0.01
3	Cyanidin-3-hexoside-epicatechin- epicatechin	0.1 ± 0.01	0.1 ± 0.01	ND	0.1 ± 0.01	0.1 ± 0.02
4	Cyanidin-3-galactoside	68.7 ± 0.5	69.3 ± 0.6	65.6 ± 0.5	68.9 ± 0.4	69.3 ± 0.6
5	Cyanidin-3-glucoside	2.6 ± 0.1	2.5 ± 0.1	2.6 ± 0.1	2.5 ± 0.1	2.6 ± 0.2
6	Cyanidin-3-arabinoside	25.0 ± 0.2	24.2 ± 0.2	26.9 ± 0.3	24.6 ± 0.2	24.2 ± 0.3
7	5-carboxypyranocyanidin-3-hexoside	0.3 ± 0.02	0.3 ± 0.01	0.3 ± 0.01	0.3 ± 0.02	0.2 ± 0.01
8	5-carboxypyranocyanidin-3-pentoside	0.1 ± 0.01	0.1 ± 0.01	0.2 ± 0.02	0.1 ± 0.01	0.1 ± 0.01
9	Cyanidin-3-xyloside	2.7 ± 0.1	3.1 ± 0.1	4.0 ± 0.2	3.2 ± 0.1	3.2 ± 0.1
Total anthocyanins, mg C3GlE */100 g FW		590.0 ± 12.4	620.5 ± 16.5	388.1 ± 8.9	614.7 ± 13.3	615.8 ± 13.7

* C3GlE: cyanidin-3-glucoside equivalents.

**Table 3:** The contents of catechins and TPACs.

Compound	“Nadzeya”	“Aron”	“Cherno-Plodnaya”	“Venisa”	“Mulatka”
Catechin, mg/100 g FW	ND ^1^	ND	Traces ^2^	ND	Traces
Epicatechin, mg/100 g FW	8.3 ± 0.2	6.3 ± 0.2	1.7 ± 0.1	2.5 ± 0.1	1.7 ± 0.1
Total proanthocyanidins, mg PCB2E/100 g FW	2524.1 ± 37.7	2198.9 ± 35.6	1395.8 ± 24.0	2212.4 ± 31.3	2476.0 ± 35.5

^1^ ND: less than 0.01 mg/100 g FW. ^2^ Traces: less than 0.1 mg/100 g FW.

**Table 4:** Identification of chokeberry flavonols using HPLC–DAD–MS.

No.	Flavonol	Rt, min	λ_max_, nm	HESI–MS^+^
1	Quercetin-dihexoside	13.4	255, 265, 354	627.25 [M + H]^+^, 465.39 [M − hexose + H]^+^, 303.10 [M − 2 hexoses + H]^+^
2	Quercetin-3-vicianoside	14.4	256, 266, 355	597.25 [M + H]^+^, 303.09 [M − vicianose + H]^+^
3	Rutin	15.5	256, 266, 355	611.25 [M + H]^+^, 465.17 [M − rhamnose + H]^+^, 303.12 [M − rutinose + H]^+^
4	Hyperoside	16.7	256, 266, 356	465.22 [M + H]^+^, 303.13 [M − galactose + H]^+^
5	Isoquercitrin	16.9	256, 266, 356	465.20 [M + H]^+^, 303.08 [M − glucose + H]^+^
6	Quercetin	26.8	255, 267, 372	303.10 [M + H]^+^

**Table 5:** Flavonol contents in chokeberries, mg/100 g FW.

No.	Flavonol	“Nadzeya”	“Aron”	“Cherno- Plodnaya”	“Venisa”	“Mulatka”
1	Quercetin-dihexoside	5.8 ± 0.1	6.3 ± 0.2	2.2 ± 0.1	3.8 ± 0.1	3.8 ± 0.1
2	Quercetin-3-vicianoside	5.7 ± 0.1	5.7 ± 0.1	3.0 ± 0.1	3.5 ± 0.1	3.4 ± 0.1
3	Rutin	12.3 ± 0.3	15.8 ± 0.3	7.0 ± 0.2	10.2 ± 0.2	9.8 ± 0.2
4	Hyperoside	16.7 ± 0.3	19.5 ± 0.3	5.7 ± 0.1	11.2 ± 0.2	10.9 ± 0.2
5	Isoquercitrin	10.0 ± 0.2	10.5 ± 0.2	3.1 ± 0.1	6.4 ± 0.1	6.1 ± 0.1
6	Quercetin	0.4 ± 0.02	0.3 ± 0.01	0.3 ± 0.01	0.1 ± 0.01	0.1 ± 0.01
	Total flavonols	50.9 ± 0.5	58.1 ± 0.6	21.3 ± 0.2	35.2 ± 0.3	34.1 ± 0.3

**Table 6:** HCA contents in chokeberries, mg/100 g FW.

HCA	“Nadzeya”	“Aron”	“Cherno- Plodnaya”	“Venisa”	“Mulatka”
Neochlorogenic acid	45.8 ± 0.2	31.6 ± 0.2	17.5 ± 0.1	15.0 ± 0.1	33.2 ± 0.2
Chlorogenic acid	81.2 ± 0.4	47.4 ± 0.3	29.2 ± 0.2	21.3 ± 0.2	43.1 ± 0.3
Cryptochlorogenic acid	9.9 ± 0.2	3.0 ± 0.1	1.3 ± 0.02	4.8 ± 0.1	6.7 ± 0.1
Total HCAs	136.9 ± 0.6	82.0 ± 0.5	48.0 ± 0.4	41.1 ± 0.3	83.0 ± 0.4

**Table 7:** Total polyphenol contents and antiradical activity against DPPH.

Cultivar	Total Polyphenols, mg GAE */100 g FW	Antiradical Activity, mM TE **/100 g FW
“Nadzeya”	1369.9 ± 27.4	4.00 ± 0.08
“Aron”	1594.5 ± 31.9	4.75 ± 0.10
“Chernoplodnaya”	747.2 ± 14.8	2.09 ± 0.04
“Venisa”	1651.5 ± 33.0	4.26 ± 0.07
“Mulatka”	1667.8 ± 33.4	4.31 ± 0.06

* GAE: gallic acid equivalents. ** TE: Trolox equivalents.

**Table 8:** Organic acid contents in chokeberries, mg/100 g FW.

Organic Acid	“Nadzeya”	“Aron”	“Cherno- Plodnaya”	“Venisa”	“Mulatka”
Malic acid	879.4 ± 17.6	927.4 ± 18.5	517.2 ± 10.3	950.0 ± 19.0	873.4 ± 17.5
Citric acid	45.1 ± 0.9	43.4 ± 0.9	35.9 ± 0.72	44.7 ± 0.9	33.7 ± 0.7
Succinic acid	91.9 ± 1.8	240.8 ± 4.8	98.2 ± 2.0	124.5 ± 2.5	111.7 ± 2.2
Quinic acid	465.2 ± 9.3	482.2 ± 9.6	396.1 ± 7.9	483.7 ± 9.7	443.2 ± 8.9
Shikimic acid	9.1 ± 0.2	8.3 ± 0.2	6.0 ± 0.1	8.5 ± 0.2	7.5 ± 0.2
Total organic acids	1490.7 ± 29.8	1702.1 ± 34.0	1053.4 ± 21.0	1611.4 ± 32.2	1469.5 ± 29.4

**Table 9:** Sugar and sorbitol contents in chokeberries, g/100 g FW.

Sugar/Sugar Alcohol	“Nadzeya”	“Aron”	“Cherno-Plodnaya”	“Venisa”	“Mulatka”
Fructose	4.89 ± 0.09	4.82 ± 0.08	4.71 ± 0.08	4.93 ± 0.09	5.12 ± 0.10
Glucose	5.08 ± 0.10	4.89 ± 0.09	4.85 ± 0.08	5.25 ± 0.10	5.32 ± 0.09
Sucrose	0.08 ± 0.005	0.01 ± 0.001	0.04 ± 0.002	ND ^1^	0.02 ± 0.002
Total sugars	10.05 ± 0.11	9.72 ± 0.10	9.60 ± 0.10	10.18 ± 0.11	10.46 ± 0.12
Sorbitol	5.71 ± 0.07	5.69 ± 0.07	5.21 ± 0.05	5.58 ± 0.05	5.61 ± 0.07

^1^ ND: less than 0.001 g/100 g FW.

**Table 10:** HPLC–DAD–MS conditions.

HPLC–DAD–MS Conditions	Anthocyanin Profile	Flavonol Profile		HCAs
LC column	Phenomenex Luna C18(2) 250 mm × 4.6 mm, 5 µm			
Mobile phase component A	1% HCOOH in H_2_O		0.1% HCOOH in H_2_O	
Mobile phase component B	1% HCOOH in acetonitrile		0.1% HCOOH in acetonitrile	
Gradient elution	0 min: 10% B 10 min: 12% B 20 min: 15% B 30–32 min: 30% B 33–45 min: 10% B	0 min: 15% B 35–40 min: 60% B 41–50 min: 15% B		0 min: 10% B 18 min: 25% B 30 min: 40% B 35 min: 60% B 36–45 min: 10% B
Flow rate	0.5 mL/min			
Column temperature	40 °C		30 °C	
Injection volume	10 µL		5 µL	
DAD wavelengths	520 nm	370 nm, 350 nm		330 nm, 275 nm
	200–700 nm	200–400 nm		
Ionization source	Heated electrospray ionization (HESI)			
Mode	Positive			Negative
Capillary voltage	3500 V			2500 V
Ion source temperature	350 °C	325 °C		275 °C
Ion transfer tube temperature	325 °C	300 °C		275 °C
Sheath gas flow rate	5.6 L/min			
Auxiliary gas flow rate	8.0 L/min			
Sweep gas flow rate	1.5 L/min			
MS scanning *m*/*z* range	150–1500			100–1000

## Data Availability

Not applicable.

## Sample Availability

Samples of the compounds are available from the authors.
